# Warm Footbaths with *Sinapis nigra* or *Zingiber officinale* Enhance Self-Reported Vitality in Healthy Adults More than Footbaths with Warm Water Only: A Randomized, Controlled Trial

**DOI:** 10.1155/2021/9981183

**Published:** 2021-07-12

**Authors:** Jan Vagedes, Silja Kuderer, Eduard Helmert, Matthias Kohl, Florian Beissner, Henrik Szöke, Stefanie Joos, Ursula Wolf

**Affiliations:** ^1^ARCIM Institute (Academic Research in Complementary and Integrative Medicine), Im Haberschlai 7, 70794 Filderstadt, Germany; ^2^Department of Neonatology, University Hospital Tuebingen, Calwerstraße 7, 72076 Tuebingen, Germany; ^3^Department of Pediatrics, Filderklinik, Im Haberschlai 7, 70794 Filderstadt, Germany; ^4^Institute of Precision Medicine, University Furtwangen, Jakob-Kienzle-Straße 17, 78054 Villingen-Schwenningen, Germany; ^5^Insula Institute for Integrative Therapy Research, Brabeckstraße 177E, 30539 Hannover, Germany; ^6^Department of Integrative Medicine, University of Pécs, Vörösmarty Utca 3, 7623 Pécs, Hungary; ^7^Institute for General Practice and Interprofessional Care, University Hospital Tuebingen, Osianderstraße 5, 72076 Tuebingen, Germany; ^8^Institute of Complementary and Integrative Medicine, University of Bern, Fabrikstrasse 8, 3012 Bern, Switzerland

## Abstract

**Objectives:**

To examine the effects of warm footbaths with thermogenic medicinal powders on vitality and heart rate variability in healthy adults. *Intervention and Outcome*. Seventeen healthy young adults (22.1 ± 2.4 years, 11 females) received three footbaths (WA: warm water only; GI: warm water and ginger; MU: warm water and mustard) in randomized order with a crossover design. We assessed vitality with the Basler Befindlichkeit questionnaire (BBS) and heart rate variability (HRV) before (*t*0), immediately after (*t*1), and 10 minutes following footbaths (*t*2). The primary outcome measure was self-reported vitality, measured via the BBS, at *t*1.

**Results:**

The primary outcome measure, self-reported vitality, was higher after GI and tended to be higher after MU compared to WA with medium effect sizes (GI vs. WA, mean difference −2.47 (95% CI −5.28 to 0.34), *p*_adj_=0.048, *d*_*adj*_ = 0.74), MU vs. WA, −2.35 (−5.32 to 0.61), *p*_adj_=0.30, *d*_*adj*_ = 0.50). At *t*2, the standard deviation of beat-to-beat intervals (SDNN) of HRV increased, and the stress index tended to decrease after all three footbath conditions with small to medium effect sizes (0.42–0.66).

**Conclusion:**

There is preliminary evidence that footbaths with thermogenic agents GI and MU may increase self-reported vitality during a short-time period with a more pronounced effect with GI. After a short follow-up, all three conditions tended to shift the autonomic balance towards relaxation. Future research should investigate these effects in clinical samples with a larger, more diverse sample size.

## 1. Introduction

Fatigue and reduced vitality are frequent symptoms in many disorders [1, 2] such as cancer, depression, or multiple sclerosis [1, 3, 4] and are also common complaints in otherwise healthy individuals [1, 4]. Self-reported vitality is defined as a positive sense of energy and aliveness [5, 6] and is influenced by both somatic and psychological factors [5]. Vitality is a marker of health status [5], which is associated with beneficial immune and antiviral responses [6] and reduced risk of cardiovascular disease [7]. Lower vitality and fatigue can origin from being overweight [4] or from physical or mental stress [1, 3]. Hancock (1995) described the problem of fatigue in daily life as follows: “We're fried by work, frazzled by the lack of time. […] No wonder one-quarter of us say we're exhausted” [1]. Direct consequences of acute stress are changes in heart rate variability (HRV) [8], a sympathetically mediated vasoconstriction, which in turn is accompanied by a drop in skin temperature [9], myocardial ischemia, and an increased risk for cardiovascular events [10]. Beyond that, low vitality is associated with a higher risk for cancer, cardiovascular disease, and an elevated mortality risk [11]. Thus, reducing perceived stress is a desirable aim in the treatment of diseases and also for healthy individuals [12].

Warm footbaths could be beneficial for increasing self-reported vitality as they induce relaxing effects [13–18] and increase comfort and the sense of well-being [14]. Associations have been reported between physical health, endogenous warmth, and well-being [19]. The application of footbaths has effects not only on distal skin temperatures, but also on perceived body warmth [20, 21] and, thus, could meaningfully impact the sense of vitality. However, the effects of footbaths on autonomic function are inconsistent and conflicting [13, 14, 16, 17, 22]. These mixed findings indicate that potential effects may depend on the experimental conditions and settings [16]. Of note, the majority of the available research focuses on footbaths with warm water only (WA). Further benefits may derive from the addition of thermogenic substances, such as ginger (*Zingiber officinale*, GI) and mustard (*Sinapis nigra*, MU), given that their active ingredients are able to penetrate skin when externally applied [20, 23–25]. Nonetheless, the effect of these substances on self-reported vitality remains unclear when added to warm footbaths.

This study was part of a larger initiative that aimed to examine the psychophysiological effects of footbaths with GI and MU. Previous research focused on short-term changes in warmth perception and skin temperature [20], as well as on the specific profiles of action of both substances during the footbaths (not yet published). The purpose of this analysis was to investigate the effects of footbaths with GI, MU, or WA on the self-reported vitality, mental state, and HRV, which adds new scientific contribution to the existing literature.

## 2. Methods

### 2.1. Study Design

This study followed a blinded randomized vehicle-controlled clinical trial with a crossover design. Participants received all three conditions in a random sequence with a washout period of at least 2 days between the different footbaths. The study was conducted at the ARCIM Institute, Germany, from October to December 2013. The design was reviewed and approved by the ethics committee of the University of Tuebingen and was recorded at the German Clinical Trials Register (DRKS) (DRKS00005350). All participants provided written informed consent before enrollment.

### 2.2. Study Population

To recruit participants, we distributed flyers and posted announcements at the school of nursing at a German hospital. Eligible participants were adults (all gender), with ages being 18–30 years old. Exclusion criteria included infectious diseases (with more than 38°C core body temperature), skin injuries in the lower legs or feet, cardiac arrhythmias, pregnancy, hypersensitivity to GI or MU products, self-reported use of medication that could influence study's outcome measures (vasoactive substances or medication with influence on heart rate variability such as sympathomimetic/sympatholytic drugs, phosphodiesterase inhibitors, or tricyclic antidepressants [26]), bronchial asthma, and insufficient knowledge of the German language. Participants were asked to provide information about their sex, age, height, and weight in order to determine their body-mass-index (BMI). Participants were also asked to refrain from consuming coffee or nicotine within three hours before each of the three footbath interventions.

### 2.3. Study Interventions

We began each footbath intervention with a brief verbal introduction (2 min). For reasons of observation and data collection, participants were provided with hospital gowns to leave the feet and lower legs uncovered. We instructed participants to sit quietly for ten minutes in order to achieve a relaxed and stable starting point prior to data collection. Following the relaxation period, participants received the footbath containing either WA, MU, or GI. We instructed them to keep their feet immersed as long as they felt comfortable. However, to minimize the potential for harm (e.g., skin irritation or burning), we did not permit participants to exceed a maximum limit of 20 minutes. We prepared all footbaths with 12 liters of water heated to 40.0 ± 0.1°C, placed within plastic tubs (water depth: 15 cm). When evaluating MU or GI, we added eighty grams of prepared powder (Caesar & Loretz GmbH, Hilden, Germany) leading to 6.67 g/l. Both powders were produced according to Good Manufacturing Practice (GMP) in compliance with the German Drug Law (AMG) and the German Good Manufacturing Regulation (Arzneimittel-und Wirkstoffherstellungsverordnung, AMWHV). After the footbaths, participants remained seated quietly for ten minutes (recovery period). We monitored the duration of footbath immersion, water and room temperature, and humidity at each session. In order to account for the circadian rhythmicity of the autonomic nervous system [27] and to achieve standardization, we conducted all footbath interventions and associated measurements between 1:30 and 6:30 pm. The mean washout period between successive footbaths was 7.7 ± 6.6 days (Min = 2, Max = 24), and the mean total time to complete all three footbath conditions was 15.4 ± 7.2 days (Min = 7, Max = 27).

### 2.4. Study Outcome Measures

The Basler Befindlichkeit questionnaire (BBS) was used to assess changes in self-reported feelings of well-being and actual status of mood. The BBS is a validated 16-item questionnaire (Cronbach's alpha 0.83 ≥ *α* ≥ 0.91) [28] that requires approximately 5 minutes for completion. Items are rated on a seven-point Likert scale (ranging from 1 = low level of activation to 7 = high level of activation) and summed according to the subscales vitality, inner balance, vigilance, and social extroversion (scale 4–28), as well as calculation of a total sum score (scale 16–112). The BBS was administered before intervention (baseline or t0), directly after immersion (postimmersion or t1), and 10 minutes following the footbath (follow-up or t2). The primary outcome measure was the BBS subscale vitality at t1. Secondary outcome measures were the BBS subscales vitality (at *t*2), inner balance, vigilance, social extroversion, and the total sum score of all items (at *t*1 and *t*2).

Other outcome measures included cardiorespiratory parameters measured with the SRM CardioScout Multi ECG System (Innovative Medical Solutions, Stuttgart, Germany) and analyzed with the HRV Scanner BioSign software (BioSign GmbH, Ottenhofen, Germany). The ECG was recorded throughout the entire intervention period (from t0 to t2). For analysis, the last 6 minutes of the baseline relaxation period, the footbath immersion, and the recovery period were taken, and of these, the last minute was discarded to ensure recoding quality and adequate duration, which resulted in five-minute segments per measurement period (*t*0, *t*1, and *t*2). These segments underwent automated t-wave detection followed by analysis of the R-R-interval time course in both time domain and frequency domain. We assessed the following time domain measures: standard deviation of beat-to-beat intervals (SDNN, ms) and root mean square of successive differences (RMSSD, ms). The SDNN is a marker of overall cardiac heartrate variability [29] and displays the aggregated modulation of the sympathetic and parasympathetic activities [30], while the RMSSD represents parasympathetic activity [29]. With respect to frequency domain measures, we calculated the ratio between low frequency (LF, 0.04–0.15 Hz) and high frequency (HF, 0.15–0.40 Hz), a parameter for assessing the sympathovagal balance [31]. From the HRV scanner calculations, we further assessed the respiratory rate (1/min) and the stress index as calculated by Baevsky [32].

### 2.5. Sample Size

We were not able to identify any published studies examining the effects of footbaths with MU or GI on the mental state or HRV. Thus, parameters needed to estimate sample size were unavailable. We selected a convenience sample of 18 participants as initial evaluation.

### 2.6. Randomization

Randomization was carried out in the presence of the study nurse at the first of the three appointments. Participants were randomly assigned to receive the three intervention conditions in different sequences, stratified by gender. Based on the study design, six different sequences were possible and were encoded by the group designations a-f (a = MU − WA − GI, b = MU − GI − WA, c = WA − GI − MU, d = WA − MU − GI, e = GI − MU − WA, and f = GI − WA − MU). We prepared three copies of each group sequence, with each being enclosed in a sealed, nontransparent envelope. Participants drew one of these opaque envelopes and the study nurse documented the group/sequence assignment. Participants were provided with study identification numbers for purposes of confidentially tracking progress over time.

### 2.7. Blinding

Participants were kept unaware to the allocated footbath sequence. To avoid unblinding and to prevent potentially biased responses because of any visual cues of the substances being used, we covered the footbaths with towels during the intervention. We further applied a room spray containing essential oils between *t*0 and *t*1 in order to diminish any olfactory hints of the ginger or mustard. Before the intervention, we verified blinding by asking each participant “what kind of substance do you smell?” (response options: MU, GI, eucalyptus, lavender, citrus, and peppermint) with multiple answers being permitted. After each intervention, we asked participants “which condition did you receive today?” and provided the response options MU, GI, or WA.

### 2.8. Statistical Analysis

All analyses were performed with R (R Core Team) [33] running in RStudio (RStudio Team) [34]. Multiple imputation by chained equations was used to replace missing values (R package: mice [35]). We set the significance level for analysis at *α* = 0.05 (two-tailed). We first applied the procedure proposed by Wellek and Blettner [36] in order to assess potential asymmetrical sequence effects due to the interaction of treatment and carryover effects. We therefore calculated the total (sum) of all three periods of the initial values (*t*0) of the primary outcome measure per subject. A nonsignificant finding in a subsequent one-factorial analysis of variance (ANOVA) with randomization group as the factor would allow us to pool the groups together for the analysis of intervention effects. Baseline demographics were reported descriptively. We analyzed the primary outcome measure (BBS vitality at *t*1) using a linear mixed effects model (R package: lme4 [37]) allowing for the footbath condition (WA, MU, and GI) as fixed effect and the subjects as random effect. The baseline measurement of the primary outcome measure (*t*0) was fitted as covariate. In the process of model selection, we calculated 95% confidence intervals (CI), the Akaike information criterion (AIC), and the Bayesian information criterion (BIC) and conducted a likelihood ratio statistic to decide whether the baseline measurements of the primary outcome measure (*t*0) or the footbath immersion duration should be considered as covariates. For the final model, we applied the method of Kenward and Roger for test statistics' approximation [38]. Post hoc comparisons of a significant main effect were based on the lmerTest package [39] with *p* values being adjusted by using a Bonferroni correction. The calculation of Cohen's *d* effect sizes was based on the covariate adjusted means and standard deviations (*d*_adj_). For all outcome measures, we calculated mean differences between the footbath conditions at *t*0, *t*1 and *t*2 as well as mean differences between *t*0 − *t*1 and *t*0 − *t*2 within each footbath condition with 95%-CI and Cohen's effect sizes (R package: effsize [40]). We analyzed baseline comparisons of the room/water temperature and humidity with one-factorial ANOVAs with condition as the factor. To test differences in the immersion times between the footbath conditions, we executed a one-factorial mixed ANOVA with condition as the fixed effect and subjects as the random effect. The success of blinding was verified using the Cochran–Mantel–Haenszel chi-squared statistics taking the total number of olfactory perceptions into account as confounder. Data was cross-checked to assure it conformed to a normal distribution.

## 3. Results

### 3.1. Study Population

**Figure 1:**
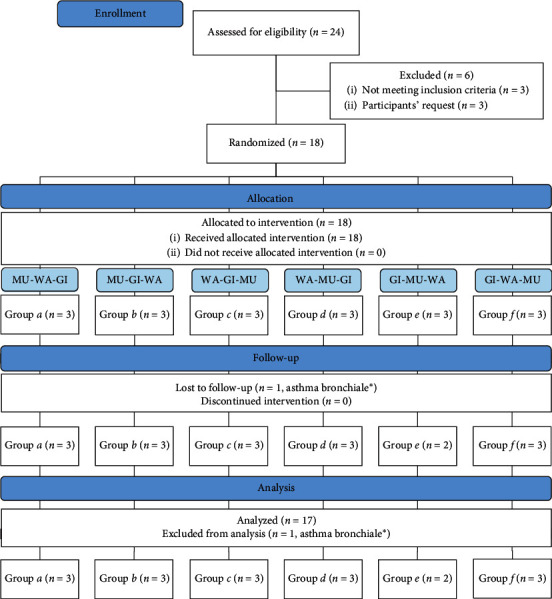
CONSORT flow diagram (WA = water only condition, GI = ginger added to water, MU = mustard added to WA, ^*∗*^same participant).

Twenty-four individuals were assessed for eligibility, of whom three (12%) did not meet the inclusion criteria (one was older than 30 years; two reported skin problems) and three (12%) decided not to participate. We randomly assigned the remaining 18 participants but subsequently excluded one (6%) from analysis upon discovering she had bronchial asthma requiring regular medication (Figure 1). The final sample (*n* = 17) consisted of eleven women (65%) and six men (35%) between 19 and 28 years of age (*m* = 22.1 ± 2.5) with an average BMI of 22.8 ± 3.7 kg/m^2^. Baseline data (Table 1) were similar between the groups.

### 3.2. Baseline Conditions

Mean starting conditions were a room temperature of 23.2 ± 0.8°C, a humidity index of 39.6 ± 7.6%, and a water temperature of 40.0 ± 0.1°C. For these parameters, no significant differences were found between the footbath conditions (room temperature: *F* (2, 48) = 2.78, *p*=0.07; humidity: *F* (2, 48) = 0.49, *p*=0.62; water temperature: *F* (2, 48) = 0.86, *p*=0.43). However, we found a significant difference with respect to participants' voluntary footbath immersion duration (*F*(2, 32) = 32.10, *p* < 0.001; MU: *m* = 11.47 ± 5.06 minutes, GI: *m* = 16.94 ± 3.54 minutes, WA: *m* = 20.00 ± 0.00 minutes). A Bonferroni-adjusted post hoc analysis revealed significant differences between all conditions and large effect sizes (WA vs. MU: *p* < 0.001, *d* = 2.38; WA vs. GI: *p* < 0.01, *d* = 0.84; MU vs. GI: *p* < 0.001, *d* = 1.06). Three participants reported coffee intake within three hours before the footbath intervention (1 in each condition). Based on the potential interaction between caffeine, self-reported vitality, mental state, and HRV parameters [41–48], these measurements were excluded from analysis and replaced with missing imputation. Approximately 6% of BBS and 12% of HRV data were missing and were imputed via MICE.

### 3.3. Analysis of Possible Carryover Effects

We detected no significant difference between the total sum for the primary outcome measure (BBS vitality) of the six different sequence groups at t0 (*F* (5, 11) = 0.57, *p*=0.72). Thus, we assumed that possible carryover effects were negligible. Randomization groups were pooled together with regard to the intervention received for the following main analysis (WA vs. MU vs. GI) (*n* = 17).

### 3.4. Model Selection

In order to determine the optimal mixed effects analysis, we compared three models (wherein condition served as fixed effect, and subjects as the random effect): model *A*, without covariates; model *B*, with baseline measurements of BBS vitality (*t*0) as covariate; and model *C*, with immersion duration as the only covariate. Model decision was based on a CI calculation (model *B*: baseline measurement: 0.35; 0.78, model *C*: immersion time: −0.21; 0.38) and on a model comparison considering the AIC (model *A*: 287.18, model *B*: 266.08, model *C*: 288.85), the BIC (model *A*: 296.84, model *B*: 277.67, model *C*: 300.44), and the likelihood ratio statistics (model B: (1) = 23.10, *p* < 0.01, model *C*: *X*_diff_^2^(0) = 0.00, *p*=1.00). Based on these results, we decided to apply model *B* (with baseline BBS vitality as covariate) for analyzing the primary outcome measure.

### 3.5. Outcomes and Estimations

#### 3.5.1. Primary Outcome: BBS Vitality at *t*1

**Figure 2:**
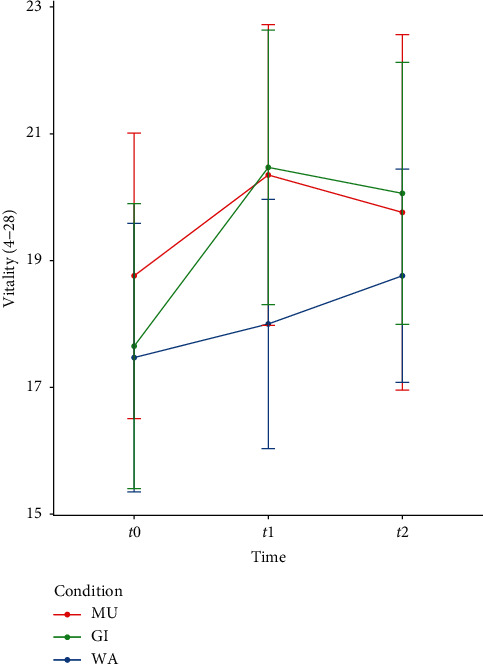
Subscale vitality of the Basler Befindlichkeit questionnaire (BBS) with means and 95% confidence intervals at *t*0, *t*1 and *t*2. Note: WA = water only condition, GI = ginger added to water, MU = mustard added to WA, *t*0 = baseline, *t*1 = after immersion, and *t*2 = follow-up.

Vitality differed significantly between the footbath conditions at t1 (*F* (2, 31) = 3.38, *p*=0.047). Post hoc analyses revealed that the covariate adjusted mean after GI (*M*_adj_ = 20.65, SD_adj_ = 3.21) was significantly higher than that of WA only (*M*_adj_ = 18.29, SD_adj_ = 3.21) (*t* (30) = −2.55, *p*_adj_=0.048, *d*_adj_ = 0.74). No significant differences and smaller effect sizes were found for the post hoc comparisons between M*X*_diff_^2^U (*M*_adj_ = 19.88, SD_adj_ = 3.23) and WA (*t* (31) = 1.70, *p*_adj_=0.30, *d*_adj_ = 0.50) and between MU and GI (*t* (31) = −0.83, *p*_adj_=1.00, *d*_adj_ = 0.24). The descriptive analysis pointed to a higher self-reported vitality after GI and MU compared to WA (Figure 2), which can be seen as a trend in the between-differences (medium effect sizes for the comparisons between WA and MU, as well as between WA and GI at *t*1, Table 2) and in the increase of the within-differences (significant increases between *t*0 and *t*1 for GI and MU, Table 3).

#### 3.5.2. Secondary Outcomes: BBS

The descriptive analysis yielded no significant differences between (Table 2) or within (Table 3) the footbath conditions over time. However, the effect sizes pointed to the trends of a sustained increase of self-reported vitality (Table 2, WA vs. GI: *d* = 0.35; Table 3, GI, t2 vs. *t*1: *d* = 0.57) and of higher increases of the total sum score (Table 3, GI, *t*1 vs. *t*0: *d* = 0.43, *t*2 vs. *t*0: *d* = 0.30) after GI.

#### 3.5.3. Secondary Outcomes: HRV

With respect to the mean between-substance differences, the footbaths did not significantly differ as a function of time (Table 4). Moreover, no differences were seen for the within-substance differences between *t*0 and *t*1 (Table 3). However, with respect to the changes between *t*0 and *t*2, the SDNN increased significantly in all footbath conditions with higher effect sizes for GI (*d* = 0.61) and MU (*d* = 0.57). Regarding the stress index, a significant reduction occurred for MU and WA between *t*0 and *t*2 (both *d* = 0.66), while for GI, a tendential reduction was seen (*d* = 0.42). For MU and WA, the trend of these changes was already apparent at *t*1. In contrast, directly after GI, the tendencies of an initial decline in the SDNN and an unchanged stress index were seen (Table 3). The changes of the RMSSD, LF/HF-Ratio and the respiration rate were less clear. The RMSSD tended to increase between *t*0 and *t*2 after all footbath conditions with a significant effect for WA (despite a small effect size) (Table 3). The between-substance differences pointed to a lower LF/HF-Ratio after GI compared to WA at *t*1 and *t*2 (0.56 ≥ *d* ≥ 0.66) (Table 4). This might be related to an unchanged ratio after WA (*d* = 0.01) and to a slightly decreased ratio after GI (*d* = 0.33) (Table 3). The within-substance differences indicated no homogenous changes of the respiration rate (Table 3). The trend of a higher respiration rate after GI and WA compared to MU might, therefore, be attributed to the differing baseline values (Table 4).

### 3.6. Success of Blinding

A total of 51 footbaths were administered as each of the 17 participants received all three conditions. At *t*0, the correct footbath thermogenic ingredient was named in nine cases (18%) (MU: *n* = 5, GI: *n* = 4). The substances most frequently mentioned as olfactory perceptions were citrus (*n* = 31) and eucalyptus (*n* = 16). We found no significant association between GI and GI olfactory perceptions (*X*^2^ (1) = 1.06, *p*=0.30) or between MU and MU olfactory perceptions (*X*^2^ (1) = 2.98, *p*=0.08), so that blinding was judged as successful at *t*0. However, when asked for the condition they received at the end of treatment (*t*2), correct answers were given in 39 cases (76%) (MU: *n* = 17, GI: *n* = 11, WA: *n* = 11).

### 3.7. Safety

A redness of the feet, which resolved within a few minutes, was observed by the study nurse and participants after all conditions. We detected no other adverse effects. Based on our decision to discontinue the footbath interventions when subjects felt uncomfortable or when they reached the time maximum, no undesired skin reactions (e.g., prolonged redness, irritation, or burning of the skin) occurred.

## 4. Discussion

The study findings demonstrated an increase in self-reported vitality directly after footbaths with GI and MU, with the highest increase after GI. After a 10-minute follow-up, all three footbath conditions (including warm water only) tended to induce autonomic changes with an increase in the SDNN and a decrease in the stress index. Interestingly, directly after the footbaths with GI, the SDNN initially decreased, and the stress index slightly increased.

These results suggest an additional effect of footbaths with GI or MU compared to footbaths with WA on self-reported vitality, but a comparable effect on HRV. The underlying mechanisms might be the skin penetration of the active ingredients of GI and MU when externally applied [23–25] and the binding to receptors of the transient receptor potential (TRP) ion channel superfamily [49–51]. The active ingredients of ginger, shogaols, activate mainly TRPV1 (TRP vanilloid 1) [49–51], which can be characterized as heat receptors [50, 52, 53]. Allyl isothiocyanate, the active ingredient of MU, in contrast, activates both TRPV1 and TRPA1 (TRP ankyrin 1) [49–51], with the latter being classified as a cold receptor [50, 52, 53]. The differing receptor activation pattern might explain the different effects of GI and MU on self-reported warmth perception as described in a previous analysis [20]. The longer-lasting warming effect of GI could be one possible explanation for the more pronounced increase in self-reported vitality. Interestingly, the ingestion of Korean red ginseng in the treatment for cold sensitivity in the hands and feet has been shown to significantly improve skin temperature but decreased self-reported vitality [54], so that warmth does not seem to be the only determinant for vitality. Furthermore, the physical effects of GI and MU might be not only attributable to the warmth increase as TRPV1 receptors are not only found in sensory neurons [49, 50, 52, 55], but also in nonneuronal tissues including blood vessels [56]. Importantly, Doering et al. reported a significant reduction in the cerebral blood flow velocity in the Arteria cerebri media after mustard footbaths, which presumably was triggered by the stimulation of thermoreceptors and the extracellular matrix [23]. Michlig et al. examined the impact of the TRP channel agonist capsaicin on the autonomic nervous system and reported a stimulation of its sympathetic branch [57]. Interestingly, shogaols are reported to have similar effects like capsaicin [58], the pungent component of chili peppers [51]. The ingestion of both ingredients stimulates catecholamine secretion [58, 59] from the adrenal medulla through beta-adrenergic stimulation of the central nervous system probably mediated by TRPV1 [51]. As MU mainly activates different channels, no comparable effect on catecholamine secretion could be observed in rats [51, 59]. The catecholamine secretion by GI supports the warming action [59] and could explain the initial sympathetic response (lower SDNN, higher stress index) directly after the footbaths. After the short follow-up (10 minutes), however, the autonomic balance shifted towards relaxation (higher SDNN, lower stress index). Similarly, Saeki observed a delayed autonomic change towards relaxation when adding the essential oil of lavender to warm footbaths [14]. The comparable HRV-changes in all three footbath conditions at the brief follow-up may indicate that the effect on the autonomic nervous system can be explained by the warmth of the water rather than by the added substances. The increase of the SDNN and RMSSD indicates an overall strengthening of the parasympathetic nervous system and a reduction of the stress level. A potential additional GI substance effect can be seen in the indicated reduction of the LF/HF-Ratio. Yao et al. reported a decrease of the ratio in a pleasant thermal environment by strengthening the vagal activity [60]. The stronger influence of GI on the LF/HF-Ratio could therefore be related to the higher influence on the heat balance [20, 21]. The LF/HF-Ratio reflects the sympathovagal balance (with higher values indicating a dominance of the sympathetic nervous system) [61]. However, due to its complex nature, the LF/HF-Ratio should be interpreted with caution [62]. In other studies, influences on the autonomic activity [13, 63] and serum cortisol levels [16, 64] with increases in relaxation have also been reported for footbaths. Water temperature was not kept constant throughout the footbath intervention, as in other studies [14, 16]. The mean temperature drop is expected to be approximately 1.6°C in 20 minutes for WA, GI, and MU [20], which means that the water temperature was approximately 38.4°C at the end of the experiment. Ishikawa conducted footbaths (40°C water temperature, 15 minutes) in 110 healthy adults (20–39 years) and observed a significant increase in SDNN (from 48 ± 19 to 60 ± 35 ms), while no changes were seen for the RMSSD or LF/HF-Ratio [30]. However, Uebaba et al. reported stress-inducing effects and a parasympathetic suppression when the water temperature reached 42°C [17]. Thus, autonomic effects might derive primarily from participants' experience of comfort from the warm (but not too hot) footbaths rather than from changes in skin temperature. In other words, positive emotions originating from higher levels of the central nervous system might trigger the observed shifts in the autonomic balance [13]. This is in accordance with the conclusion of Frank et al. who emphasized that skin surface temperature mainly contributes to self-reported thermal comfort and not to autonomic responses [65]. Furthermore, the higher increase in self-reported vitality after GI and MU may be related to the stronger stimulation (thermal and chemical stimulation) compared to WA (only thermal stimulation). The advantage of GI (higher and longer lasting increase of self-reported vitality) over MU could be linked to partial differing reaction pattern of the autonomic nervous system after GI. The primary sympathetic reaction induced a counterregulation with a secondary parasympathetic dominance. Thus, stimulation and activation may induce feelings of being vital and alive, in conjunction with perceptions of also being relaxed.

Despite the randomized controlled study design, this trial has several limitations. First, a potential limitation of the study is the relatively small sample size. This was further limited by the fact that three participants reported coffee intake within three hours before the footbath intervention (1 in each condition). We decided to impute these measurements as caffeine potentially affects self-reported vitality, mental state, and HRV parameters. Second, we were able to blind the participants at *t*0 by applying a room spray containing essential oils; however, at *t*2, the majority named the correct ingredient. This unblinding was probably triggered by the substance-specific effects of GI and MU and may have influenced the assessment of the self-reported parameters at *t*2. In addition, the room spray may have had an influence on the outcome measures of the study. In the field of aromatherapy, essential oils are used to instill vitality and to improve the perception of stress and the measurable stress index [66]. Due to the small sample size, the findings must be interpreted cautiously and do not allow for a statement towards generalizability. That said, the study does contribute to the greater understanding of short-term self-reported vitality and how it can be induced through GI and MU. Further studies are required to confirm the observed effects, to examine the potential therapeutic value of such thermogenic substances when added to footbaths, and to clarify the potential modes of action. Moreover, future studies should analyze the exact powder compositions (e.g., with liquid chromatography-tandem mass spectrometry for ginger [67] and time-of-flight mass spectrometry for mustard products [68]) to improve chemical standardization and to clarify the specific effects of single components. Additional outcome measures, such as the serum cortisol level, could aid in gaining a better understanding of the physiological effects of footbaths on vitality. Third, the study focused on healthy young adult individuals. Clinical studies will be important to conduct with patient-participants diagnosed with primary physical or psychiatric disorder who suffer from secondary fatigue to determine if footbaths could be an appropriate treatment option to increase self-reported vitality, improve the quality of life, and impact disease etiology and pathogenesis. As part of such investigations, the amount of thermogenic substance supplementation and duration of such footbaths also merit further investigation.

## 5. Conclusion

In conclusion, we were able to demonstrate a short-term increase in self-reported vitality amongst healthy young adult participants after warm footbaths. The effects differed between footbaths with warm water only and footbaths that were supplemented with ginger or mustard, with the highest effects observed after ginger. The HRV data pointed to a substance-independent autonomic change with a shift to an increased parasympathetic activity at the brief follow-up. It is possible that the heightened self-reported vitality induced by such supplemented footbaths might contribute to a higher quality of self-reported relaxation that is at once characterized by vitality and relaxation. Thus, footbaths could potentially serve as an accessible, natural, economical therapeutic adjunct for those suffering from fatigue associated with chronic illness by helping to induce states of relaxation and to increase self-reported vitality when thermogenic substances are added.

## Figures and Tables

**Table 1:** Baseline (*t*0) characteristics of study's participants.

Group (number)	a (*n* = 3)	b (*n* = 3)	c (*n* = 3)	d (*n* = 3)	e (*n* = 2)	f (*n* = 3)
Footbath sequence	MU − WA − GI	MU − GI − WA	WA − GI − MU	WA − MU − GI	GI − MU − WA	GI − WA − MU

*Demographic*						
Age, years	21.00 ± 2.65	22.67 ± 2.08	23.00 ± 1.73	24.00 ± 3.61	19.50 ± 0.71	21.67 ± 1.15
BMI, kg/m^2^	22.87 ± 1.89	24.24 ± 8.43	21.06 ± 0.90	23.16 ± 4.40	23.90 ± 0.18	21.87 ± 1.15
Sex, female *n* (%)	2 (66.67)	2 (66.67)	2 (66.67)	2 (66.67)	1 (50.00)	2 (66.67)

*Basler Befindlichkeit questionnaire (BBS)*						
Vitality^a^	18.67 ± 3.39	19.33 ± 4.44	16.56 ± 5.20	17.33 ± 2.40	15.33 ± 5.89	19.67 ± 3.64
Intrapsychic balance^a^	22.33 ± 3.64	21.33 ± 3.50	22.67 ± 4.42	23.67 ± 1.41	22.50 ± 3.73	23.56 ± 3.32
Vigilance^a^	19.44 ± 3.13	18.11 ± 4.96	16.67 ± 3.20	19.78 ± 4.68	18.83 ± 7.19	19.33 ± 3.35
Social extroversion^a^	17.44 ± 4.39	15.89 ± 3.69	15.11 ± 3.37	20.00 ± 4.42	19.67 ± 3.50	17.00 ± 3.28
Total sum score^b^	77.89 ± 11.32	74.67 ± 10.46	71.00 ± 12.27	80.78 ± 8.94	76.33 ± 19.48	79.56 ± 10.96

*Cardiorespiratory parameters*						
SDNN, ms	47.24 ± 10.87	49.06 ± 24.77	47.44 ± 9.94	52.33 ± 22.85	66.50 ± 19.14	46.43 ± 14.82
RMSSD, ms	24.42 ± 7.08	27.65 ± 15.46	29.18 ± 4.33	30.79 ± 16.58	47.85 ± 20.24	28.55 ± 10.16
LF/HF-ratio	7.03 ± 6.07	3.08 ± 1.98	4.23 ± 4.46	2.85 ± 1.11	2.21 ± 1.35	2.42 ± 1.75
Stress index, pts.	182.48 ± 91.74	234.78 ± 195.89	196.09 ± 90.90	181.18 ± 119.00	98.45 ± 57.35	176.30 ± 102.36
Respiration rate, 1/min	16.48 ± 2.08	18.90 ± 4.54	17.02 ± 1.23	14.48 ± 2.58	14.75 ± 2.22	19.35 ± 0.66

Notes: data are means ± SD of all participants; if not, otherwise indicated. WA = water only condition, GI = ginger added to water, MU = mustard added to WA. ^a^[4; 28]; ^b^[16; 112].

**Table 2:** Mean values for Basler Befindlichkeit questionnaire (BBS) at *t*0, *t*1, and *t*2 and between-substance differences as a function of time.

	Mean ± SD				Mean difference (95% CI); |Cohen's *d*|		
		WA	MU	GI	∆WA − MU	∆WA − GI	∆MU − GI
Vitality^a^	*t*0	17.47 ± 4.12	18.76 ± 4.38	17.65 ± 4.37	−1.29 (−4.27; 1.68), 0.30	−0.18 (−3.15; 2.79); 0.04	1.12 (−1.94; 4.18); 0.26
	*t*1	18.00 ± 3.82	20.35 ± 4.61	20.47 ± 4.21	−2.35 (−5.32; 0.61); 0.56	−2.47 (−5.28; 0.34); 0.61	−0.12 (−3.20; 2.97); 0.03
	*t*2	18.76 ± 3.27	19.76 ± 5.45	20.06 ± 4.02	−1.00 (−4.17; 2.17); 0.22	−1.29 (−3.86; 1.27); 0.35	−0.29 (−3.65; 3.06); 0.06

Intrapsychic balance^a^	*t*0	22.29 ± 4.30	22.76 ± 2.41	23.00 ± 3.30	−0.47 (−2.93; 1.99); 0.14	−0.71 (−3.39; 1.98); 0.18	−0.24 (−2.26; 1.79); 0.08
	*t*1	22.82 ± 2.96	23.65 ± 2.69	23.12 ± 2.96	−0.82 (−2.80; 1.15); 0.29	−0.29 (-2.36; 1.77); 0.10	0.53 (−1.45; 2.50); 0.19
	*t*2	22.29 ± 3.51	23.71 ± 2.89	23.00 ± 3.54	−1.41 (−3.66; 0.84); 0.44	−0.71 (−3.17; 1.76); 0.20	0.71 (−1.55; 2.96); 0.22

Vigilance^a^	*t*0	19.76 ± 4.13	18.59 ± 4.61	17.71 ± 4.27	1.18 (−1.88; 4.24); 0.27	2.06 (−0.88; 4.99); 0.49	0.88 (−2.22; 3.99); 0.20
	*t*1	19.18 ± 3.41	19.47 ± 5.46	19.06 ± 3.80	−0.29 (−3.50; 2.91); 0.06	0.12 (−2.41; 2.64); 0.03	0.41 (−2.89; 3.71); 0.09
	*t*2	18.71 ± 3.79	18.59 ± 5.87	18.06 ± 4.52	0.12 (−3.36; 3.59); 0.02	0.65 (−2.27; 3.56); 0.16	0.53 (−3.14; 4.20); 0.10

Social extroversion^a^	*t*0	17.53 ± 4.98	17.59 ± 3.57	17.06 ± 3.67	−0.06 (−3.10; 2.98); 0.01	0.47 (−2.59; 3.53); 0.11	0.53 (−2.00; 3.06); 0.15
	*t*1	17.24 ± 4.31	17.82 ± 3.52	17.76 ± 4.24	−0.59 (−3.34; 2.17); 0.15	−0.53 (−3.51; 2.46); 0.12	0.06 (−2.67; 2.78); 0.02
	*t*2	17.29 ± 4.03	17.47 ± 3.81	17.88 ± 3.98	−0.18 (−2.92; 2.56); 0.05	−0.59 (−3.39; 2.21); 0.15	−0.41 (−3.13; 2.31); 0.11

Total sum score^b^	*t*0	77.06 ± 13.70	77.65 ± 10.83	75.47 ± 11.84	−0.59 (−9.24; 8.06); 0.05	1.59 (−7.37; 10.54); 0.12	2.18 (−5.75; 10.11); 0.19
	*t*1	77.24 ± 10.36	81.29 ± 13.19	80.41 ± 11.07	−4.06(−12.36; 4.25); 0.34	−3.18(−10.67; 4.31); 0.30	0.88 (−7.63; 9.40); 0.07
	*t*2	77.06 ± 11.09	79.53 ± 14.03	79.00 ± 11.74	−2.47(−11.32; 6.38); 0.20	−1.94 (−9.92; 6.04); 0.17	0.53 (−8.52; 9.58); 0.04

Notes: WA = water only condition, GI = ginger added to water, MU = mustard added to WA, *t*0 = baseline, *t*1 = after immersion, *t*2 = follow-up, CI = confidence intervals. ^a^[4; 28]; ^b^[16; 112].

**Table 3:** Change in study's outcome measures from *t*0 to *t*1 (∆*t*1 − *t*0) and *t*0 to *t*2 (∆*t*2 − *t*0) as a function of footbath condition.

		∆*t*1 − *t*0			∆*t*2 − *t*0		
		Diff	95% CI (L; H)	|*d*|	Diff	95% CI (L; H)	|*d*|
*Basler Befindlichkeit questionnaire (BBS)*							
Vitality^a^	WA	0.53	(−0.61; 1.67)	0.13	1.29	(−0.40; 2.99)	0.35
	MU	**1.59**	(0.40; 2.78)	0.35	1.00	(−0.35; 2.35)	0.20
	GI	**2.82**	(0.19; 5.45)	0.66	2.41	(−0.05; 4.87)	0.57
Intrapsychic balance^a^	WA	0.53	(−0.86; 1.92)	0.14	0.00	(−1.37; 1.37)	0.00
	MU	0.88	(−0.80; 2.57)	0.35	0.94	(−0.82; 2.70)	0.35
	GI	0.12	(−1.51; 1.74)	0.04	0.00	(−2.30; 2.30)	0.00
Vigilance^a^	WA	−0.59	(−2.04; 0.87)	0.16	−1.06	(−2.83; 0.71)	0.27
	MU	0.88	(−1.00; 2.76)	0.17	0.00	(−2.26; 2.26)	0.00
	GI	1.35	(−1.50; 4.21)	0.33	0.35	(−2.42; 3.13)	0.08
Social extroversion^a^	WA	−0.29	(−1.97; 1.38)	0.06	−0.24	(−2.14; 1.67)	0.05
	MU	0.24	(−1.33; 1.80)	0.07	−0.12	(−1.83; 1.60)	0.03
	GI	0.71	(−1.06; 2.48)	0.18	0.82	(−0.84; 2.49)	0.22
Total sum score^b^	WA	0.18	(−4.26; 4.62)	0.01	0.00	(−5.49; 5.49)	0.00
	MU	3.65	(−0.53; 7.82)	0.30	1.88	(−3.48; 7.25)	0.15
	GI	4.94	(−1.91; 11.79)	0.43	3.53	(−4.04; 11.10)	0.30

*Cardiorespiratory parameters*							
SDNN, ms	WA	1.82	(−6.02; 9.67)	0.09	**9.08**	(2.59; 15.56)	0.49
	MU	4.40	(−5.64; 14.44)	0.29	**8.33**	(0.31; 16.36)	0.57
	GI	-3.79	(−10.71; 3.12)	0.23	**11.75**	(3.15; 20.34)	0.61
RMSSD, ms	WA	0.82	(−3.93; 5.57)	0.06	**3.92**	(0.03; 7.81)	0.27
	MU	1.68	(−4.25; 7.61)	0.14	3.75	(−1.70; 9.21)	0.29
	GI	1.35	(−3.05; 5.75)	0.10	4.71	(−0.36; 9.78)	0.34
LF/HF-Ratio	WA	−0.11	(−1.49; 1.27)	0.05	0.03	(−1.08; 1.13)	0.01
	MU	−0.72	(−2.10; 0.66)	0.27	−0.34	(−2.33; 1.66)	0.12
	GI	−1.39	(−3.59; 0.81)	0.38	−1.24	(−3.33; 0.85)	0.33
Stress index, pts.	WA	−22.80	(−48.21; 2.60)	0.21	−**61.60**	(−106.30;-16.91)	0.66
	MU	−35.45	(-99.77; 28.86)	0.33	−**61.55**	(−112.53;-10.57)	0.66
	GI	0.63	(−29.42; 30.67)	0.00	−43.23	(−91.05; 4.60)	0.42
Respiration rate, 1/min	WA	0.56	(−0.57; 1.70)	0.18	−0.40	(−1.68; 0.88)	0.13
	MU	0.59	(−0.36; 1.53)	0.23	0.59	(−0.63; 1.81)	0.24
	GI	0.91	(−0.57; 2.39)	0.36	0.14	(−1.26; 1.53)	0.06

Notes: data are means ± SD of all participants. WA = water only condition, GI = ginger added to water, MU = mustard added to WA, *t*0 = baseline, *t*1 = after immersion, *t*2 = follow-up, Diff = mean difference, CI = confidence intervals, *L* = lower confidence limit, *H* = upper confidence limit, *d* = Cohen's d effect size). ^a^[4; 28]; ^b^[16; 112]. Bold indicates confidence intervals that do not contain zero.

**Table 4:** Mean values for cardiorespiratory parameters at *t*0, *t*1, and *t*2 and between-substance differences as a function of time.

	Mean ± SD				Mean difference (95% CI); |Cohen's *d*|		
		WA	MU	GI	∆WA − MU	∆WA − GI	∆MU − GI
SDNN, ms	*t*0	52.91 ± 20.58	46.81 ± 16.31	52.13 ± 17.53	6.09 (−6.91; 19.09); 0.33	0.78 (−12.59; 14.15); 0.04	−5.31 (−17.14; 6.52); 0.31
	*t*1	54.73 ± 17.83	51.21 ± 14.50	48.34 ± 14.91	3.52 (−7.86; 14.89); 0.22	6.40 (−5.10; 17.89); 0.39	2.88 (−7.40; 13.15); 0.20
	*t*2	61.99 ± 16.42	55.15 ± 12.57	63.87 ± 21.08	6.84 (−3.41; 17.08); 0.47	−1.89 (−15.12; 11.34); 0.10	−8.73 (−20.96; 3.51); 0.50

RMSSD, ms	*t*0	30.20 ± 14.17	28.72 ± 13.59	32.40 ± 14.81	1.48 (−8.22; 11.18); 0.11	−2.20 (−12.33; 7.93); 0.15	−3.69 (−13.62; 6.25); 0.26
	*t*1	31.02 ± 13.89	30.40 ± 9.88	33.76 ± 12.75	0.62 (−7.84; 9.08); 0.05	−2.74 (−12.05; 6.58); 0.21	−3.36 (−11.35; 4.63); 0.29
	*t*2	34.12 ± 14.33	32.47 ± 12.15	37.11 ± 12.94	1.65 (−7.64; 10.94); 0.12	−2.99 (−12.53; 6.55); 0.22	−4.64 (−13.41; 4.13); 0.37

LF/HF-Ratio	*t*0	3.68 ± 2.29	3.60 ± 3.45	3.88 ± 4.96	0.08 (−1.98; 2.14); 0.03	−0.20 (−2.94; 2.54); 0.05	−0.29 (−3.28; 2.71); 0.07
	*t*1	3.57 ± 1.58	2.87 ± 1.44	2.49 ± 1.71	0.70 (−0.35; 1.76); 0.46	1.08 (−0.07; 2.23); 0.66	0.38 (−0.72; 1.49); 0.24
	*t*2	3.71 ± 2.06	3.26 ± 2.06	2.64 ± 1.76	0.45 (−0.99; 1.89); 0.22	1.06 (−0.27; 2.40); 0.56	0.62 (−0.72; 1.96); 0.32

Stress index, pts.	*t*0	175.75 ± 115.04	197.06 ± 119.96	175.92 ± 131.30	−21.31(−103.42; 60.81); 0.18	−0.17 (−86.47; 86.13); 0.00	21.14(−66.75; 109.03); 0.17
	*t*1	152.95 ± 99.45	161.60 ± 89.76	176.54 ± 134.93	−8.65 (−74.87; 57.56); 0.09	−23.60(−106.69; 59.50); 0.20	−14.94(−95.48; 65.59); 0.13
	*t*2	114.15 ± 66.23	135.50 ± 52.80	132.69 ± 65.22	−21.36 (−63.29; 20.57); 0.36	−18.54 (−64.47; 27.38); 0.28	2.81 (−38.72; 44.34); 0.05

Respiration rate, 1/min	*t*0	17.39 ± 3.42	16.20 ± 2.95	17.26 ± 2.79	1.19 (−1.04; 3.43); 0.37	0.14 (−2.05; 2.32); 0.04	−1.06 (−3.06; 0.95); 0.37
	*t*1	17.96 ± 2.70	16.79 ± 2.08	18.17 ± 2.30	1.17 (−0.52; 2.86); 0.48	−0.21 (−1.97; 1.55); 0.08	−1.38 (−2.91; 0.16); 0.63
	*t*2	17.00 ± 2.48	16.79 ± 1.84	17.40 ± 1.78	0.21 (−1.32; 1.74); 0.10	−0.40 (−1.91; 1.11); 0.19	−0.61 (−1.87; 0.66); 0.34

Notes: WA = water only condition, GI = ginger added to water, MU = mustard added to WA, *t*0 = baseline, *t*1 = after immersion, *t*2 = follow-up, CI = confidence intervals.

## Data Availability

The data used to support the findings of this study are available from the corresponding author upon request.
